# Detection of Echovirus-18 in Children Suspected With SARS-CoV-2 Infection With Multisystem Inflammatory Syndrome: A Case Report From India

**DOI:** 10.3389/fpubh.2022.897662

**Published:** 2022-07-22

**Authors:** Mallika Lavania, Rajlakshmi Viswanathan, Sumit Dutt Bhardwaj, Jitendra S. Oswal, Nutan Chavan, Manohar Shinde, Savita Katendra

**Affiliations:** ^1^Enteric Viruses Group, Indian Council of Medical Research (ICMR)-National Institute of Virology, Pune, India; ^2^Bacteriology Group, Indian Council of Medical Research (ICMR)-National Institute of Virology, Pune, India; ^3^National Influenza Center Indian Council of Medical Research (ICMR)-National Institute of Virology, Pune, India; ^4^Bharati Vidyapeeth (Deemed to be) Medical College and Hospital Pune, Pune, India

**Keywords:** SARS-CoV-2, pediatric patients, Echovirus-18, MIS-C, COVID-19

## Abstract

There have been several reports across the globe regarding the presentation of a severe multi-system hyperinflammatory syndrome, resembling Kawasaki disease (KD), in the pediatric population during the SARS-CoV-2 pandemic. The exact pathophysiology is still unclear; however, children typically demonstrate multi-organ dysfunction and less respiratory system involvement compared to adults. The limited literature is available at present for the identification and management of such patients. In this study, we investigated four cases in children ages 11–15 years that fulfilled the case definition for the pediatric multi-system inflammatory syndrome. All were found negative for SARS-CoV-2 from oropharyngeal swabs and stool. As they were having symptoms of diarrhea, tests for bacterial and enteric viral infections were performed after SARS-CoV-2 testing. Molecular analysis revealed that all the children were infected with enterovirus (Echovirus-18). Early and exact diagnosis is vital for timely, effective, and potentially life-saving management of such cases.

## Introduction

The clinical presentation and severity of severe acute respiratory syndrome coronavirus 2 (SARS-CoV-2) infection are variable in the pediatric age group with a predominance of asymptomatic cases, or mild symptoms of the respiratory or gastrointestinal system (GI). In pediatric patients with SARS-CoV-2, the prevalence of GI symptoms, including diarrhea, vomiting, and abdominal pain, ranges up to 88–90%. These symptoms can develop before, during, or after the onset of respiratory symptoms (1–3). A new inflammatory condition termed multisystem inflammatory syndrome in children (MIS-C) has been noted, which includes features of shock, cardiac dysfunction, multiorgan failure, and even some features of Kawasaki disease (4). The amount of information available about the clinical presentation and epidemiologic characteristics of children with MIS-C is minimal and changes daily. The ages of affected children (*n* = 70) in a case series from the UK (5), Italy (6), France, and Switzerland (7, 8) ranged from 2–to 16 years, with the majority having no underlying comorbidities. The majority had a 4-day fever, and the most common presenting symptoms were gastrointestinal symptoms (59/70 = 84 percent), such as vomiting, abdominal pain and/or diarrhea; mucocutaneous symptoms resembling KD, such as conjunctivitis and rash; and neurologic findings, such as headache, irritability, and encephalopathy.

We report a case series of four children between the ages of 11–15 years, who were suspected cases of SARS-CoV-2 infection. The cases were part of an ongoing study for the investigation of fecal shedding of SARS-CoV-2. The Ethics Committee of both participating institutions approved this study (NIV/IEC/June/2020/D-14 dated June 24, 2020).

## Description of the Cases

Four children between 11 and 15 years of age were admitted to the hospital, over a week, with similar clinical presentations, i.e., fever, vomiting, diarrhea, abdominal pain, difficulty breathing, shock, and myocardial dysfunction (Table 1). They were considered suspected cases of SARS-CoV-2 infection with MIS-C. One child was a known case of Fanconi's anemia, who was on regular blood transfusions and oral chelation, while the others did not have any comorbidities. The children had no recent travel history and had received age-appropriate immunizations per the national immunization schedule. The mean duration of illness before admission to the hospital was 5 days (range 3–10 days). None of the close contacts of the four cases had any symptoms of or was positive for SARS-CoV-2 infection. Throat swabs and stool samples were collected upon admission for SARS-CoV-2 testing, with throat swabs collected again after 3 days. All samples were negative for SARS-CoV-2. At the time of admission, none of the family members were vaccinated for SARS-CoV-2. Due to the clinical presentation of diarrhea, vomiting, and abdominal pain, testing was performed for common enteric viruses [i.e., rotavirus A (RVA), norovirus gr I and II, astrovirus, adenovirus, and enterovirus] and common bacterial pathogens (i.e., diarrheagenic *E.coli, Salmonella* spp., *Shigella* spp., and *Vibrio cholerae*).

**Table 1:** Demographic and clinical characteristics of reported cases with multisystem inflammatory syndrome in children (MIS-C).

**Case**	**Age/sex**	**Clinical course**	**Date of admission**	**Duration of treatment**	**Date of discharge**	**Co morbidity**	**Investigations**	**Laboratory investigations**			**Clinical management**	**Response to the treatments**
								**SARS-CoV-2**	**Bacterial Testing**	**Enteric Viruses testing**		
1	11/F	Moderate fever, abdominal pain, diarrhea, hypovolemic shock, mycardial dysfunction	23.06.2020	9 days	02.07.2020	None	Anemia, deranged liver function, elevated CRP, d-Dimer, ferritin, fibrinogen, interleukin-6, procalcitonin, sterile blood culture, tropical virus/bacteria panel negative	Negative	Negative	Negative	IV fluids, ionotropic support, mechanical ventilation, antibiotics, hydrocortisone, followed by methylprednisolone, low molecular weight heparin,IV albumin, oral aspirin	Recovered
2	12/M	Moderate fever, abdominal pain, diarrhea, blanching rash over trunk and lower limbs, hypotensive shock, mycocardial dysfunction	11.06.2020	19 days	30.06.2020	None	Anemia, deranged liver function, elevated CRP, d-dimer, ferritin, interleukin-6 procalcitonin, sterile blood culture, tropical virus/bacteria panel negative	Negative	Negative	Positive (Echovirus-18)	IV fluids, high flow nasal oxygen followed by mechanical ventilation, antibiotics, dopamine, noradrenaline, methylprednisolone, Intravenous immunoglobulin	Recovered
3	14/F	High grade fever, vomiting diarrhea, hypotensive shock flowed by focal seizure, persistent hemodynamic instability, multi organ failure	05.06.2020	10 days	15.06.2020	Fanconi's Anemia	Anemia, thrombocytopenia, metabolic acidosis Hyperproteinaemia, deranged liver function, mycocardial dysfunction, hemophagocytosis, haemorrhagic encephalitis, elevated CRP, d-Dimer, ferritin, interleukin-6, sterile blood culture, tropical virus/bacteria panel negative	Negative	Negative	Positive (Echovirus-18)	IV fluids, platelet and packed cell transfusions, mechanical ventilation, noradrenaline, dobutamine, benzodiazepine followed by levetiracetam vasopressin, hydrocortisone, pulse methylprednisolone, antibiotics, low molecular weight heparin. IVIG could not be procured	Death
4	15/F	High grade fever, abdominal pain, diarrhea, lethargy, hypotensive shock	02.06.2020	7 days	09.06.2020	None	Metabolic acidosis. Hypoproteineimia, deranged liver function, elevated CRP, d-Dimer, ferritin, interleukin-6 sterile blood culture, tropical virus/bacteria panel negative	Negative	Negative	Positive (Echovirus-18)	IV fluids, mechanical ventilation, noradrenaline, hydrocortisone, antibiotics, low molecular weight heparin. IVIG could not be procured	Recovered

The fecal samples were screened for RVA by antigen-capture ELISA (Premier Rotaclone, Meridian Bioscience, Inc.) as per the manufacturer's instructions. The viral nucleic acids were extracted from 30% suspensions in phosphate buffer saline (PBS) buffer (pH 7.2-7.4) using spin columns (Qiagen, Hilden, Germany) as per the manufacturer's instructions. One part of the stool sample preserved in the Cary Blair transport medium (HiMedia Laboratories, Mumbai) was used for culture and molecular identification.

Detection of norovirus GI, GII, human astrovirus, RVA, human adenovirus, and sapovirus was done by using Multiplex Real-Time PCR for FTD Viral gastroenteritis (Fast Track Diagnostics, Luxembourg). Conventional PCR was performed for the detection of enterovirus (9). Partial VP1 viral capsid gene region was amplified using primers AN88 and AN89 (10) and sequencing was performed using BigDye Termination Ready Reaction Mix (Applied Biosystems) on an ABI Prism 3,700 DNA Analyser (Applied Biosystems). The sequence identity of EV strains was determined through the BLAST search Tool (http://www.ncbi.nlm.nih.gov/blast).

Standard protocols were adopted for the isolation and identification of common bacterial diarrheal pathogens (11). For diarrheagenic *E. coli*, three to six lactose fermenting colonies and up to three non-lactose fermenting colonies from MacConkey agar plates were selected for testing by conventional procedures. Identification of *E. coli* isolates was confirmed by the VITEK^®^ 2 COMPACT automated microbial identification system (BioMérieux, Inc. Hazelwood, USA) using VITEK 2 GN cards (BioMérieux, Marcy-l'Ètoile, France). These isolates were re-plated on Trypticase Soy Agar, from which three isolated colonies were resuspended in phosphate-buffered saline (PBS) for further extraction of genomic DNA using a QIAamp DNA kit (Qiagen, USA). Polymerase chain reaction assays were performed for diarrheagenic *E. coli* using AccuPrime *Taq* DNA Polymerase System (Thermo Fisher Scientific) primers and the same PCR conditions as described earlier (12–14).

## Results

The throat and stool samples both showed negative results for SARS-CoV-2 by qRT-PCR. ELISA for Rotavirus A was negative. All four stool samples tested negative for SARS-CoV-2, as well as Norovirus GI, GII, Human Astrovirus, Rotavirus A, and Human Adenovirus. The samples were also negative for the common bacterial pathogens tested.

**Figure 1:**
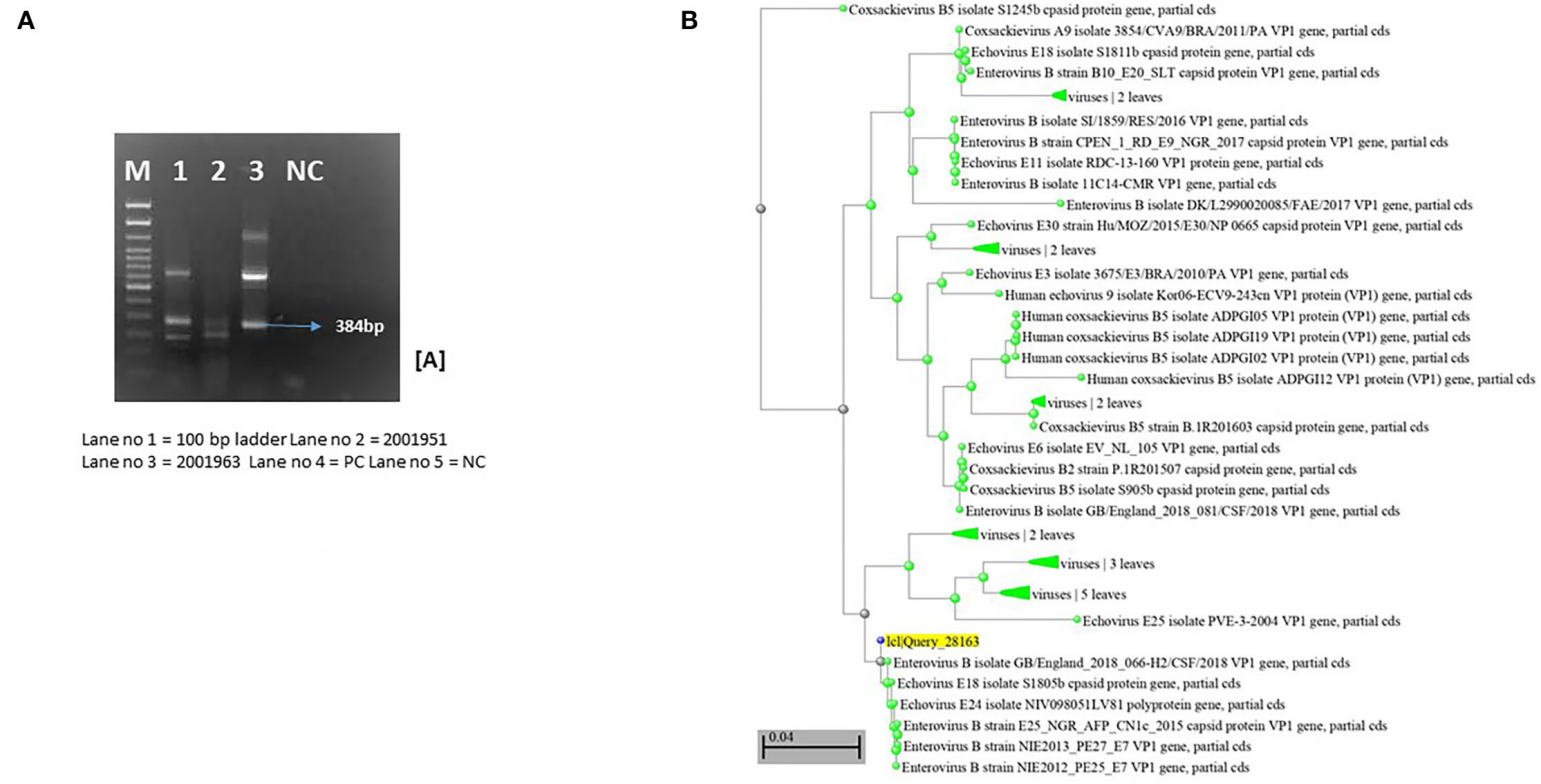
Molecular characterization of Enterovirus strains based on PCR and sequencing. **(A)** Nested PCR amplification of VP1 region of enterovirus. **(B)** Phylogenetic analysis of Enterovirus strains based on VP1 identified in cases. Study strains are highlighted in yellow. Scale indicates genetic distance.

Stool samples of three patients showed positivity for the VP 1 region of Enteroviruses, which was confirmed by semi-nested RT-PCR. Sequencing and phylogenetic analyses of two strains revealed a genotype distributed to Echovirus 18 (E18) (Figure 1), closely related to E18 isolate S1805b capsid protein gene. We were unable to get high-quality sequences from the third sample. The case with Fanconi's anemia succumbed, while the other three recovered successfully.

## Discussion

Pune has been one of the most affected cities in India during the SARS-CoV-2 pandemic. As noted worldwide, we started seeing patients with MIS-C soon after the first peak in Pune in June 2020. The cases in the present report, presented with fever, gastrointestinal symptoms, cardiac dysfunction, and shock were similar to reports from other regions. The inflammatory markers like D-dimer, serum ferritin, and interleukin-6 were elevated and the patients responded well to steroids and immunoglobulin therapy. The only death could be associated with the underlying co-morbidity of Fanconi's anemia.

There are few studies from Asia on MIS-C. With the widespread community transmission of SARS-CoV-2, patients with less typical clinical presentations, such as GI symptoms including nausea, vomiting, diarrhea, and abdominal pain, are being considered for possible SARS-CoV-2 infection. In our study, we were unable to differentiate between the symptoms of MIS-C from those associated with SARS-CoV-2 infection.

Human echovirus 18 (E-18) is a member of the enterovirus B species. It is most commonly known to cause aseptic meningitis (15). The virus is also associated with epidemic diarrhea in infants, neonatal sepsis, exanthema, and leukoencephalitis (16). Echovirus 18 most commonly affects children <1-year-old (17). A study done by Zhang et al. (18), reported the two novel echoviruses 18 recombinants associated with hand-foot-and-mouth disease. There are also a few reports about the presence of Echovirus-18 from India (19, 20).

A limitation of our study is that we could not isolate the virus. However, while isolation remains the gold standard for detection, molecular diagnostics are commonly used to confirm E-18 in patients with symptoms.

## Conclusion

Based on the clinical presentation severity, response to treatment, and laboratory findings, we hypothesize that E-18 could be a possible cause of MIS-C. However, larger studies are needed to confirm this hypothesis. Treating physicians need to remain alert to the possibility of other pathogens in the differential diagnosis. Even if SARS-CoV-2 is screened as a priority, testing for other pathogens depending on the clinical presentation and endemicity should be initiated as early as possible, especially in children. This will ensure timely and effective management of the potential E-18 cases.

## Data Availability Statement

The original contributions presented in the study are publicly available, and deposited at GenBank, under accession numbers: BankIt2602366 1951 ON985268, BankIt2602366 1963 ON985269.

## Ethics Statement

The studies involving human participants were reviewed and approved by ICMR-National Institute of Virology, Pune. Written informed consent to participate in this study was provided by the participants' legal guardian/next of kin.

## Author Contributions

ML and RV significantly contributed to conception, design, and literature review. ML, RV, and SB contributed to drafting of the manuscript and critical revision of the final version. JO contributed to providing the clinical details of the patients. NC, MS, and SK did the experimental work. All authors contributed to the article and approved the submitted version.

## Funding

This case report is a part of another study, which is funded by ICMR-National Institute of Virology, Pune, Maharashtra, India.

## Conflict of Interest

The authors declare that the research was conducted in the absence of any commercial or financial relationships that could be construed as a potential conflict of interest.

## Publisher's Note

All claims expressed in this article are solely those of the authors and do not necessarily represent those of their affiliated organizations, or those of the publisher, the editors and the reviewers. Any product that may be evaluated in this article, or claim that may be made by its manufacturer, is not guaranteed or endorsed by the publisher.
